# Extensive genomic reshuffling involved in the karyotype evolution of genus *Cerradomys* (Rodentia: Sigmodontinae: Oryzomyini)

**DOI:** 10.1590/1678-4685-GMB-2020-0149

**Published:** 2020-11-13

**Authors:** Camilla Bruno Di-Nizo, Malcolm Andrew Ferguson-Smith, Maria José de J. Silva

**Affiliations:** 1 Instituto Butantan, Laboratório de Ecologia e Evolução, São Paulo, SP, Brazil.; 2 Cambridge Resource Centre for Comparative Genomics, Department of Veterinary Medicine, University of Cambridge, Cambridge, United Kingdom.

**Keywords:** Chromosomal evolution, GTG-banding, Oryzomyini, ZOO-FISH

## Abstract

Rodents of the genus *Cerradomys* belong to the tribe Oryzomyini and present high chromosome variability with diploid numbers ranging from 2n=46 to 60. Classical cytogenetics and fluorescence *in situ* hybridization (FISH) with telomeric and whole chromosome-specific probes of another Oryzomyini, *Oligoryzomys moojeni* (OMO), were used to assess the karyotype evolution of the genus. Results were integrated into a molecular phylogeny to infer the hypothetical direction of chromosome changes. The telomeric FISH showed signals in telomeres in species that diverged early in the phylogeny, plus interstitial telomeric signals (ITS) in some species from the most derived clades (*C. langguthi,* *C. vivoi*, *C. goytaca*, and *C. subflavus*). Chromosome painting revealed homology from 23 segments of *C. maracajuensis* and *C. marinhus* to 32 of *C. vivoi*. Extensive chromosome reorganization was responsible for karyotypic differences in closely related species. Major drivers for genomic reshuffling were *in tandem* and centric fusion, fission, paracentric and pericentric inversions or centromere repositioning. Chromosome evolution was associated with an increase and decrease in diploid number in different lineages and ITS indicate remnants of ancient telomeres. Cytogenetics results corroborates that *C. goytaca* is not a junior synonym of *C. subflavus* since the karyotypic differences found may lead to reproductive isolation.

## Introduction

*Cerradomys* is a rodent genus of the tribe Oryzomyini, distributed in open vegetations of South America from northeastern Brazil to southeastern Bolivia and northwestern Paraguay. It was previously described as a subgenus of *Oryzomys* (*Oryzomys subflavus*) (Weksler *et al.*, 2006; Percequillo, 2015). From 1981 to 2002, different karyotypes were described suggesting that the genus was not monotypic (Maia and Hulak, 1981; Almeida and Yonenaga-Yassuda, 1985; Svartman and Almeida, 1992; Andrades-Miranda *et al.*, 2002), and this was confirmed later using molecular and morphological studies (Bonvicino and Moreira, 2001; Langguth and Bonvicino, 2002; Bonvicino, 2003). Then, *Oryzomys subflavus* became *Oryzomys* gr. *subflavus* and, in 2006, this group of species was raised to the genus category *Cerradomys* (Weksler *et al.*, 2006).

Currently, eight species are formally described and cytogenetic information has been an important identifying tool: *Cerradomys akroai* (2n=60, FN=74), *C. goytaca* (2n=54, FN=66), *C. langguthi* (2n=50, 49, 48, and 46, FN=56)*, C. maracajuensis* (2n=56, FN=58)*, C. marinhus* (2n=56, FN=54)*, C. scotti* (2n=58, FN=70 and 72)*, C. subflavus* (2n=54, 55 and 56, FN=62-64) and *C. vivoi* (2n=50, FN=64) (Svartman and Almeida, 1992; Langguth and Bonvicino, 2002; Bonvicino, 2003; Percequillo *et al.*, 2008; Tavares *et al.*, 2011; Bonvicino *et al.*, 2014).

Besides the cytotaxonomic importance, cytogenetics reveals substantial chromosomal variation mainly due to Robertsonian rearrangements, pericentric inversion and sex chromosome polymorphisms. This makes the genus an excellent group for studies of karyotype evolution (Maia and Hulak, 1981; Almeida and Yonenaga-Yassuda, 1985).

It has been shown that classic cytogenetic studies can fail to detect interspecific chromosomal homologies in groups with great chromosomal variability, such as rodents of the tribe Oryzomyini, and that chromosome painting studies provide the required resolution (Ferguson-Smith *et al.*, 1998). Thus, such studies were able to detect syntenic segments and shed light on the rearrangements occurring throughout the chromosomal evolution of this tribe (Nagamachi *et al.*, 2013; Di-Nizo *et al.*, 2015; Suárez *et al.*, 2015; Oliveira Da Silva *et al.*, 2017). These studies become even more informative when linked to a phylogeny, since they allow the recovery of possible trajectories of chromosomal changes (Di-Nizo *et al.*, 2015; Sotero-Caio *et al.*, 2015; Suárez *et al.*, 2015). Until now, molecular cytogenetic studies of the *Cerradomys* genus have been scarce (Nagamachi *et al.*, 2013) and its karyotype evolution remains to be explored.

The aim of this work is to investigate chromosomal homologies among *Cerradomys* species and to infer the rearrangements that have occurred during the karyotype evolution of the genus. To achieve these goals, we have performed classic cytogenetics, FISH with telomeric probes and chromosome painting using *Oligoryzomys moojeni* (OMO, 2n=70) whole chromosome probes. *Oligoryzomys* is another genus of the tribe Oryzomyini that belongs to a sister clade of *Cerradomys* (Weksler *et al.*, 2006); both lineages diverged approximately 5 Mya (Leite *et al.*, 2014). In addition, we performed molecular phylogenetic analyses to infer the hypothetical polarity of chromosome changes.

## Material and Methods

### Chromosome preparation and classical cytogenetics

Samples comprise 10 individuals referred to here as *Cerradomys marinhus* (CMARI); *C. maracajuensis* (CMARA); *C. akroai* (CAK); *C. scotti* (CSC); *C. langguthi* (CLA); *C. vivoi* (CVI); *C. goytaca* (CGO) and *C. subflavus* (CSU) (Table 1).

**Table 1:** Specimens analyzed in this work.

Species	Voucher	Locality	2n	FN	Sex	FISH OMO	Phylogeny
*C. marinhus* (CMARI)	CRB1835	Cocos, BA	56	54	M	X	X
*C. maracajuensis* (CMARA)	MN71687	Parque Nacional Emas, GO	56	58	M	X	X
*C. akroai* (CAK)	MZUSP30347	Uruçuí-Una, PI	60	76	M	-	X
*C. scotti* (CSC)	MJJS189	Serra das Galés, GO	58	72	F	X	X
*C. langguthi* (CLA)	JFV474	Piracuruca, PI	46	56	M	X	X
*C. vivoi* (CVI)	BIO555	Mucugê, BA	50	64	M	X	-
*C. goytaca* (CGO)	NPM933	Restinga de Jurubatiba, RJ	54	66	F	X	X
*C. subflavus* (CSU)	CIT2053	Itirapina, SP	56	64	M	-	X
	CIT1396	Rio Claro, SP	55	63	F	-	X
	DQM059	Serra da Canastra, MG	54	62	M	X	-

The animals surveyed by the authors were live trapped under ICMBio licences (numbers 11603-1 and 24003-4) of Instituto Chico Mendes de Conservação da Biodiversidade. Some specimens were captured by collaborators under their respective licenses (Table S1). Animals were euthanized according to “Guidelines for Animal use” (Sikes *et al.*, 2011) under permission of the Comissão de Ética para Uso de Animais do Instituto Butantan (CEUAIB 1151/13).

Skins, skulls and partial skeletons of *C. maracajuensis*, *C. marinhus* and *C. langguthi* were deposited at the Museu Nacional da Universidade Federal do Rio de Janeiro (MN); *C. akroai*, *C. scotti*, *C. vivoi* and *C. subflavus* were deposited in the Museu de Zoologia da Universidade de São Paulo (MZUSP) and *C. goytaca* was deposited at the Núcleo de Pesquisa em Ecologia e Desenvolvimento Sócio-Ambiental de Macaé (NPM). Cell suspensions or fibroblast cells are deposited in the Laboratório de Ecologia e Evolução do Instituto Butantan.

Metaphases of *C. marinhus*, *C. maracajuensis*, *C. vivoi,* and *C. subflavus* with 2n=54 were obtained *in vitro* from fibroblast cell culture (Freshney, 1986), and metaphases of *C. akroai*, *C. scotti*, *C. goytaca*, *C. langguthi*, and *C. subflavus* with 2n=55 and 2n=56 were obtained *in vivo* from spleen and bone marrow (Ford and Hamerton, 1956). All samples were analyzed (at least 30 metaphases from each individual) using conventional Giemsa staining, CBG-banding (Sumner, 1972) and GTG-banding (Seabright, 1971).

### Fluorescence *in situ* Hybridization (FISH)

Fluorescence *in situ* hybridization with telomeric probes (TTAGGG)_n_ labeled with Fluorescein isothiocyanate (FITC) was carried out on all samples following the recommended protocol (Telomere PNA FISH Kit, Code No. K5325, DAKO). Slides were counterstained with 4’,6-Diamidine-2’-phenylindole dihydrochloride (DAPI) with antifade mounting medium Vectashield. Metaphases were analyzed with an Axiophot fluorescence microscope (Carl Zeiss) using the software ISIS (Metasystem) that can overlap images of filters for DAPI and FITC. In some cases, metaphases were analyzed in an Axioskop 40 epifluorescence microscope (Carl Zeiss) equipped with the AxionVision software and propidium iodide (PI) was added to the fluorescence antifade solution (0.5 μL/mL) to visualize chromosomes.

Chromosome painting was performed on metaphases of one representative of each species - *C. marinhus*, *C. maracajuensis*, *C. scotti*, *C. langguthi*, *C. vivoi*, *C. goytaca* and *C. subflavus* with 2n=54 - (Table 1), except for *C. akroai* and *C. subflavus* (2n=55 and 56), which lacked sufficient metaphases.

Twenty specific single painting probes from *Oligoryzomys moojeni* with 2n=70, FN=72 obtained by fluorescence-activated chromosome sorting (FACS) were used (OMO Xa, OMO 1–8, 11, 16, 17, 25–30, 33, and 34; see Di-Nizo *et al.*, 2015). The probes were made by degenerate oligonucleotide-primed polymerase chain reaction (DOP-PCR) and labeled with biotin-16-dUTP (Telenius *et al.*, 1992; Yang *et al.*, 1995). FISH was performed according to previous studies (Yang *et al.*, 1995; Di-Nizo *et al.*, 2015), with probes detected with avidin-FITC. Metaphases were analyzed with specific filters for DAPI and FITC in a Zeiss Axiophot fluorescence microscope.

### Phylogenetic analyses

Molecular phylogenetic analyses based on the partial mitochondrial cytochrome *b* (cyt *b*) gene were performed to infer chromosome evolution and rearrangements within *Cerradomys* lineages.

DNA was extracted from liver or muscle with Chelex 5% (Bio-Rad) (Walsh *et al.*, 1991). Polymerase chain reaction (PCR) was performed in a thermal cycler (Eppendorf Mastercycler ep Gradient, Model 5341) using primers MVZ05 and MVZ16 (Irwin *et al.*, 1991; Smith and Patton, 1993) with conditions following Suárez-Villota *et al.* (2017). Sequencing was conducted using BigDye (DNA “Big Dye Terminator Cycle Sequencing Standart,” Applied Biosystems) and an ABI PRISM 3100 Genetic Analyzer (Applied Biosystems) and submitted to a comparative similarity search on BLAST (Basic Local Alignment Search Tool) before the alignment. Alignments were performed using Muscle (Edgar, 2004) implemented in Geneious 7.1.7 (Biomatters) (Kearse *et al.*, 2012). GenBank access numbers are provided in Table S1.

The cytochrome *b* matrix was composed of 733 bp with 30 terminal taxa (Table S1) using *Hylaeamys megacephalus* and *Neacomys amoenus* as outgroup (*sensu* Weksler *et al.*, 2006). At least two reference sequences of each *Cerradomys* species were extracted from GenBank, including holotypes and paratypes (Table S1).

Maximum Likelihood (ML) analysis was performed using Treefinder (Jobb, 2011) and nodal support was calculated using nonparametric bootstrapping (Felsenstein, 1985), with 1000 pseudoreplicates. Bayesian Inference (BI) analysis was performed with MrBayes 3.04b (Ronquist and Huelsenbeck, 2003). Markov chains were started from a random tree and run for 1.0 × 10^7^ generations with sampling every 1000th generation. The stationary phase was checked using Tracer 1.6 (Rambaut *et al.*, 2014). Sample points prior to the plateau phase were discarded as burn in, and the remaining trees were combined to find the maximum *a posteriori* estimated probability of the phylogeny. Branch supports were estimated with Bayesian posterior probabilities.

## Results

### Classical cytogenetic data

**Figure 1:**
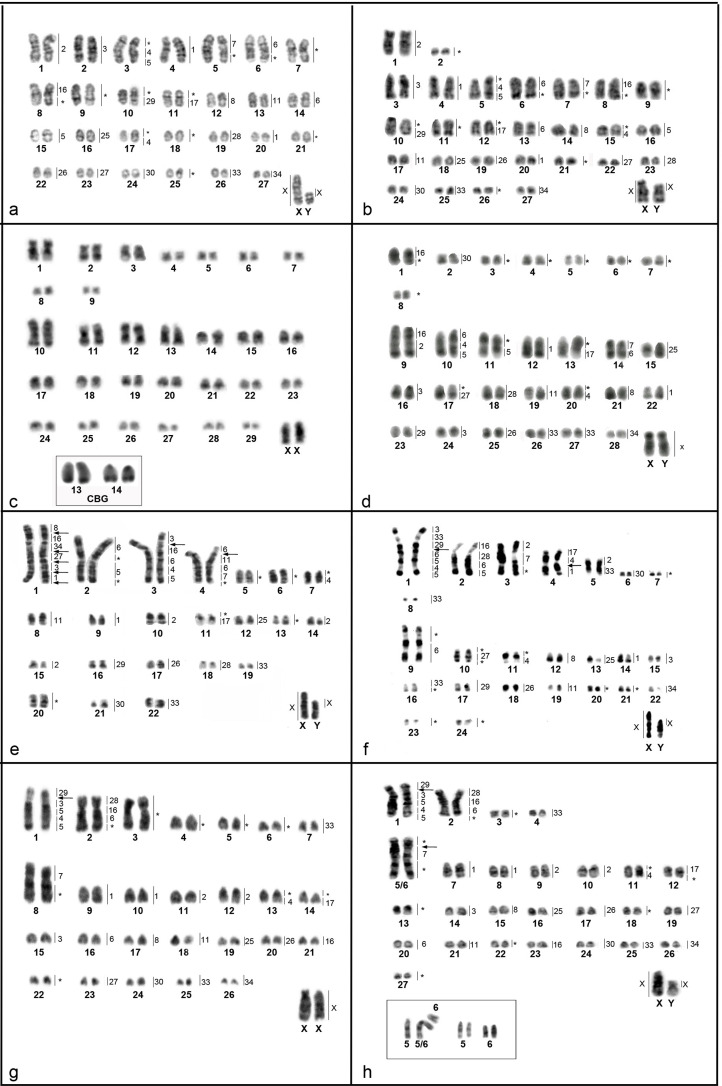
DAPI or GTG-banding of Cerradomys species: (a) C. marinhus – 2n=56, FN=54; (b) C. maracajuensis – 2n=56, FN=58; (c) C. akroai – 2n=60, FN=76, inset: CBG of pairs 13 and 14; (d) C. scotti – 2n=58, FN=72; (e) C. langguthi – 2n=46, FN=56; (f) C. vivoi – 2n=50, FN=64; (g) C. goytaca – 2n=54, FN=66 and (h) C. subflavus – 2n=54, FN=62; inset: pairs 5 and 6 involved in centric fusion in specimens with 2n=55 and 2n=56, respectively. Except for C. akroai, hybridization pattern of OMO probes are indicated beside the chromosomes. Arrows indicate ITS. *Represent regions not hybridized by any OMO probes.

Classical cytogenetics showed *Cerradomys marinhus* (CMARI) with 2n=56, FN=54 (Figure 1a); *Cerradomys maracajuensis* (CMARA) (Figure 1b) with 2n=56, FN=58; *Cerradomys akroai* (CAK) (Figure 1c) with 2n=60, FN=76; *Cerradomys scotti* (CSC) (Figure 1d) with 2n=58, FN=72; *Cerradomys langguthi* (CLA) (Figure 1e) with 2n=46, FN=56; *Cerradomys vivoi* (CVI) (Figure 1f) with 2n=50, FN=64; *Cerradomys goytaca* (CGO) (Figure 1g) with 2n=54, FN=66 and three different diploid numbers for *Cerradomys subflavus* (CSU) (Figure 1h): (i) 2n=56, FN=64; (ii) 2n=55, FN=63 and (iii) 2n=54, FN=62.

Differences in the three *C. subflavus* karyotypes concerned pairs 5 and 6: karyotype (i) showed pair 5 subtelocentric and pair 6 acrocentric; karyotype (ii) showed a large submetacentric (5/6), one subtelocentric (5) and one acrocentric (6); and karyotype (iii) showed one large metacentric pair that corresponds to pairs 5 and 6 (Figure 1h).

Patterns of CBG-banding are described here for the first time for *C. marinhus*, *C. maracajuensis*, *C. akroai* and *C. goytaca*. In *C. marinhus*, CBG-banding showed signals of heterochromatin in the centromeric region of all autosomes, in the proximal region of X and in the distal region of Y (not shown). *Cerradomys maracajuensis* showed constitutive heterochromatin in the centromeric region of autosomes, in the short arm of X and in the whole Y (not shown). Regarding *C. akroai*, CBG-banding showed a subtle signal at the pericentromeric region of autosomes. In addition, two autosomal pairs (possibly pairs 13 and 14), presented C-positive signals in the distal regions while the sex chromosomes did not have heterochromatic blocks (Figure 1c). CBG-banding in *C. goytaca* showed a weak signal, and the presence of constitutive heterochromatin was evident in the smaller pairs. The X chromosome was heterochromatic in the proximal region and the Y in the distal region (not shown).

In all species studied, GTG-banding allowed the recognition of homologues (Figure 1).

### FISH with telomeric probes

**Figure 2:**
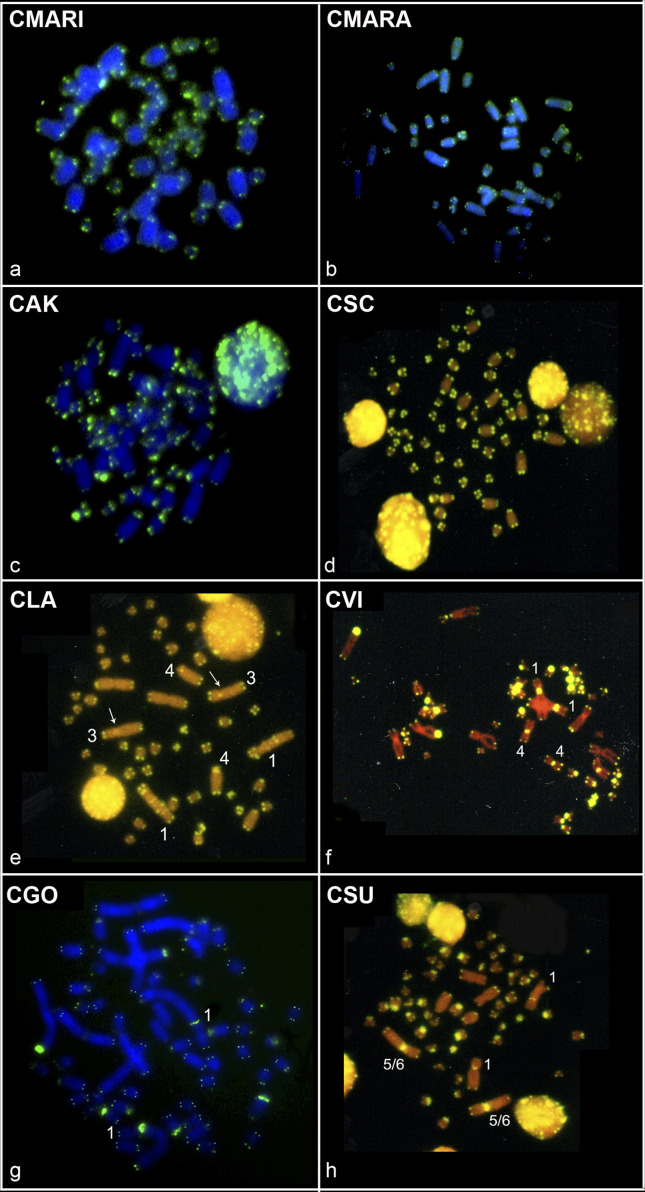
FISH with telomeric probes in Cerradomys species: (a) C. marinhus – 2n=56, FN=54; (b) C. maracajuensis – 2n=56, FN=58; (c) C. akroai – 2n=60, FN=76; (d) C. scotti – 2n=58, FN=72; (e) C. langguthi – 2n=46, FN=56 (arrows indicate the ITS signals observed in pair 3); (f) C. vivoi – 2n=50, FN=64; (g) C. goytaca – 2n=54, FN=66 and (h) C. subflavus – 2n=54, FN=62. Chromosomes that bear ITS are indicated.

FISH with telomeric probes showed signals exclusively on telomeric regions of *C. marinhus, C. maracajuensis*, *C. akroai* and *C. scotti* (Figure 2a-d).

In addition to telomeric regions, positive signals at interstitial sites (ITS) were observed in the remaining species: *C. langguthi* showed multiple ITSs in the largest submetacentric pair (1) and pairs 3 and 4 showed signals in the pericentromeric position (Figure 2e); *C. vivoi* showed interstitial telomeric sites (ITS) in the centromeres of pairs 1 and 4 (Figure 2f); *C. goytaca* in the pericentromeric region of pair 1 (Figure 2g); and *C. subflavus* with 2n=54 and 2n=55 showed ITS in the pericentromeric regions of pairs 1 and 5/6 (Figure 2h) while the sample with 2n=56 showed ITS only in pair 1.

### Chromosome painting with *Oligoryzomys moojeni* (OMO) probes

Chromosome painting using OMO probes revealed 23 homologous segments in metaphases of *C. marinhus* and *C. maracajuensis*, 26 in *C. scotti*, 31 in *C. langguthi*, 32 in *C. vivoi,* and 27 in *C. goytaca* and *C. subflavus* with 2n=54 (Table 2). Hybridization of different OMO probes and equivalent G-bands are shown in Figure 1. Some chromosomes were not hybridized by any probe (assigned with asterisks in Figure 1), probably because the probes used did not include all chromosome pairs of *O. moojeni*.

**Table 2:** Homologous segments detected by chromosome painting with *Oligoryzomys moojeni* (OMO) probes in metaphases of seven *Cerradomys* species: *C. marinhus* (CMARI), *C. maracajuensis* (CMARA), *C. scotti* (CSC), *C. langguthi* (CLA), *C. vivoi* (CVI), *C. goytaca* (CGO) and *C. subflavus* (CSU).

OMO	CMARI	CMARA	CSC	CLA	CVI	CGO	CSU
OMO 1	4 20	4 20	12 22	1 (distal) 9	4q (distal) 14	9 10	7 8
OMO 2	1	1	9 (distal)	10 14 15	3p 5p	11 12	9 10
OMO 3	2	3	16 24	1 (interstitial) 3p	1p 15	1p 15	1p 14
OMO 4	3 (interstitial) 17 (distal)	5 (interstitial) 15 (distal)	10 (interstitial) 20 (distal)	3 (interstitial) 7 (distal)	1 (interstitial) 4 (proximal) 11 (distal)	1 (interstitital) 13 (distal)	1 (interstitial) 11 (distal)
OMO 5	3 (distal) 15	5 (distal) 16	10 (distal) 11 (distal)	3 (distal) 2q	1q (two egions) 2q (distal)	1q (two regions)	1q (two regions)
OMO 6	6 (proximal) 14	6 (proximal) 13	10 (proximal) 14 (distal)	2q (proximal) 3(pericentromeric) 4 (interstitial) 4p	1 (interstitial) 2 (proximal) 9 (distal)	2q (interstitial) 16	2q (interstitial) 20
OMO 7	5 (proximal)	7 (proximal)	14 (proximal)	4 (interstitial)	3q (proximal)	8 (interstitital)	5 (proximal)
OMO 8	12	14	21	1 (distal)	12	17	15
OMO 11	13	17	19	4 (proximal) 8	19	18	21
OMO 16	8 (proximal)	8 (proximal)	1p 9 (proximal)	3 (proximal) 1 (proximal)	2p (distal)	2q (proximal) 21	2q (proximal) 23
OMO 17	11 (distal)	12 (distal)	13 (distal)	11 (distal)	4p	14 (distal)	12 (proximal)
OMO 25	16	18	15	12	13	19	16
OMO 26	22	19	25	17	18	20	17
OMO 27	23	22	17 (distal)	1 (interstitial)	10 (interstitial)	23	19
OMO 28	19	23	18	18	2p (proximal)	2p	2p
OMO 29	10 (distal)	10 (distal)	23	16	1 (interstitial) 17	1p (distal)	1p (distal)
OMO 30	24	24	2	21	6	24	24
OMO 33	26	25	26 27	19 22	1 (interstitial) 5q 8 16 (proximal)	7 25	4 25
OMO 34	27	27	28	1 (proximal)	22	26	26
OMO Xa	X, Yp	X, Yp	X	X, Y (proximal)	X, Y (proximal)	X	X, Y (proximal)
**Total**	**23**	**23**	**26**	**31**	**32**	**27**	**27**

Considering the 20 OMO probes, 11 hybridized to whole chromosomes of *C. marinhus* and *C. maracajuensis*; eight hybridized to whole chromosomes of *C. scotti;* five painted whole chromosomes of *C. langguthi;* six painted whole chromosomes of *C. vivoi;* and seven hybridized to whole chromosomes of *C. goytaca* and *C. subflavus* (Table 2).

Besides, four paints produced a single signal in one region or chromosome arm of *C. marinhus*, *C maracajuensis*, *C. scotti*, *C. goytaca* and *C. subflavus* and five probes hybridized to one chromosomal region or arm of *C. langguthi* and *C. vivoi* (Table 2).

Four OMO probes hybridized to more than one chromosome or chromosome region of *C. marinhus* and *C maracajuensis;* seven probes hybridized to more than one pair of *C. scotti;* eight probes painted more than one pair or region of *C. vivoi*, *C. goytaca* and *C. subflavus* and nine probes painted more than one pair or region of *C. langguthi* (Table 2).

**Figure 3:**
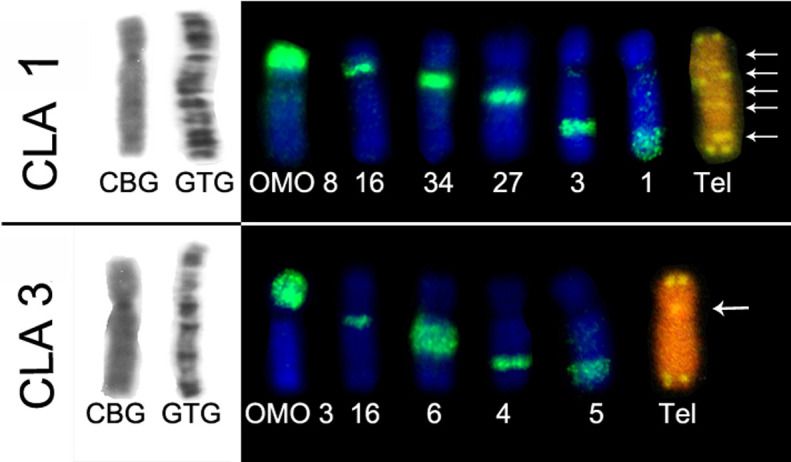
CBG and GTG-banding pattern and hybridizations with OMO and telomeric probes in chromosomes CLA 1 and CLA 3 of C. langguthi. Arrows indicate ITS.

**Figure 4 f4:**
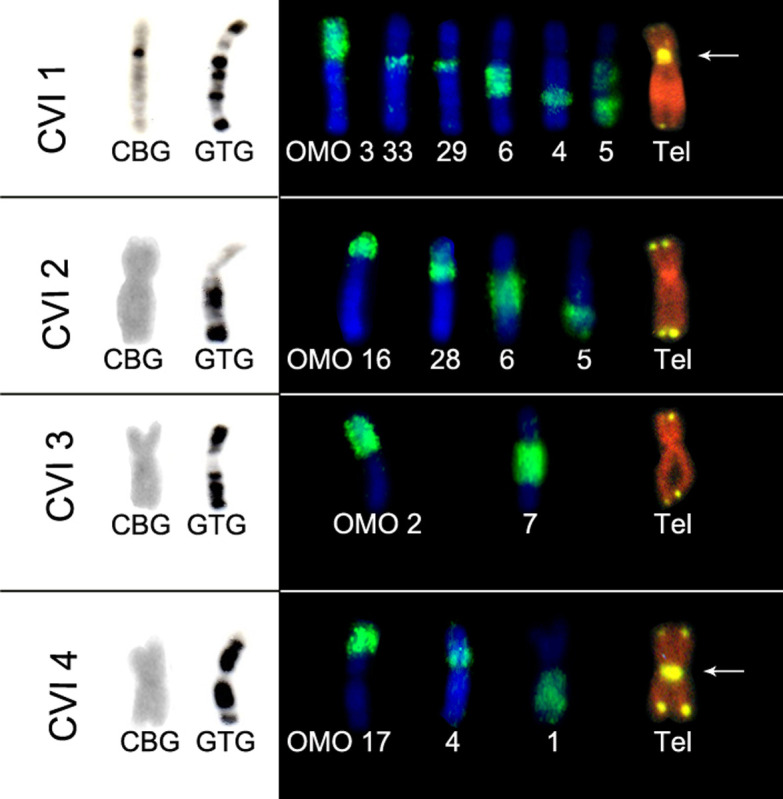
CBG and GTG-banding pattern and hybridizations with OMO and telomeric probes in four pairs of C. vivoi (CVI 1 to 4). Arrows indicate ITS.

**Figure 5 f5:**
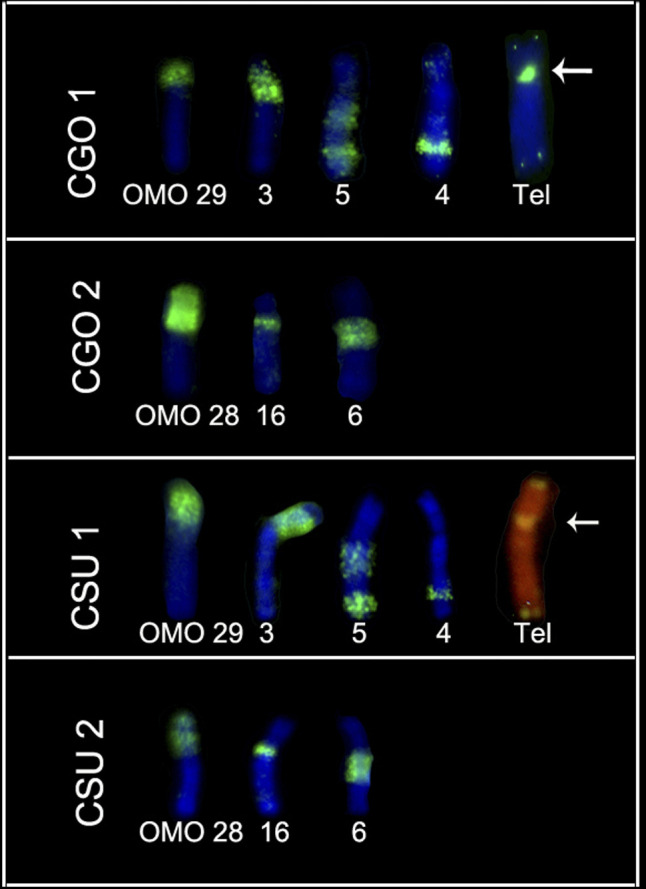
Hybridizations of OMO and telomeric probes in pairs 1 and 2 of C. goytaca and C. subflavus. Arrow indicates ITS.

Associations of OMO probes were observed: in *C. langguthi,* six OMO probes hybridized in chromosome CLA 1 and five in CLA 3 (Figure 3). Twelve probes hybridized to the four largest pairs of *C. vivoi* (Figure 4). In addition, four probes painted CGO 1 and CSU 1, and three probes painted CGO 2 and CSU 2 (Figure 5).

**Figure 6:**
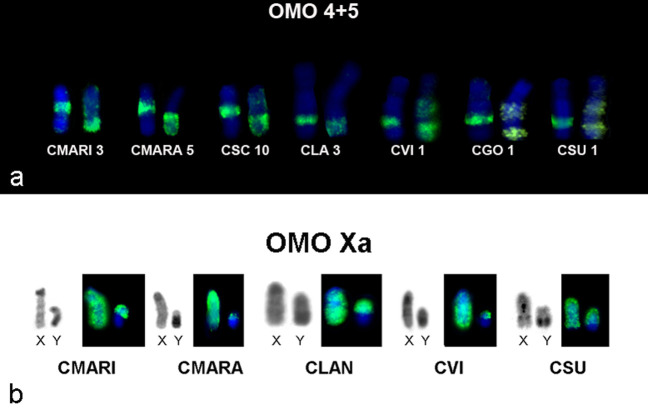
OMO probe results. (a) Associations between OMO probes 4 and 5 detected in all Cerradomys species studied; (b) Homologies of sex chromosomes of Cerradomys. CBG-banding (on the left) and chromosome painting using OMO Xa probe (on the right).

Association of probes OMO 4 and OMO 5 was observed in all species, and in *C. vivoi, C. goytaca* and *C. subflavus* two regions of the same chromosome were painted with OMO 5 probe (Figure 6a).

Sex chromosome OMO Xa probe painted the whole X and the euchromatic region of the Y chromosome in all males studied (Figure 6b).

### Phylogenetic relationships

**Figure 7:**
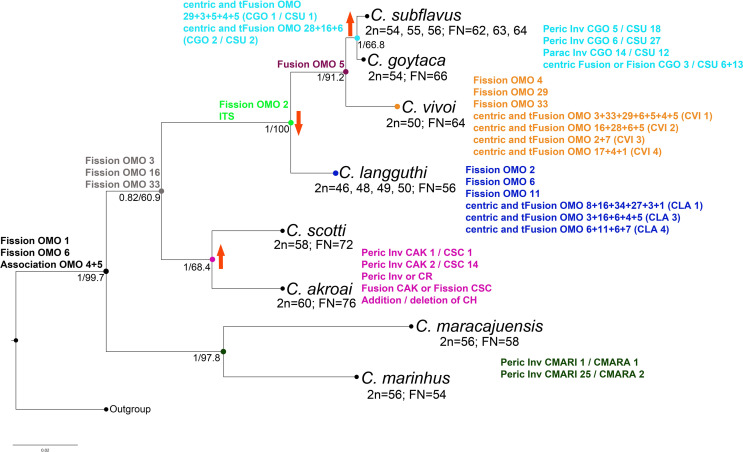
Phylogenetic relationships of Cerradomys based on cyt-b matrix and Maximum Likelihood (ML) analyses. Values in the nodes represent Bayesian posterior probability and ML bootstrap, respectively. Rearrangements detected by chromosome painting, GTG and CBG-banding are plotted. Arrows indicate increase or decrease in diploid number. Abbreviations: pericentric inversion (peric inv), paracentric inversion (parac inv), centromere repositioning (CR), constitutive heterochromatin (CH), in tandem fusion (tFusion) and interstitial telomeric signal (ITS).

Both Bayesian Inference (BI) and Maximum Likelihood (ML) analyses recovered the same topology and *Cerradomys* as monophyletic (BPP = 1.0, bootstrap = 99.7) (Figure 7; Figure S1). *C. marinhus* (2n=56, FN=54) and *C. maracajuensis* (2n=56, FN=58) were recovered as the sister group of all the other species with high support (BPP = 1.0, bootstrap = 97.8), followed by a clade with weak support (BPP = 0.82, bootstrap = 60.9) composed of the sister species *C. akroai* (2n=60, FN=76) and *C. scotti* (2n=58, FN=72) (BPP = 1.0, bootstrap = 68.4). Next clade includes the remaining species (BPP = 1.0; bootstrap = 100): *Cerradomys langguthi* (2n=46, FN=56) and its sister clade that includes *C. vivoi* (2n=50, FN= 64) and the closely related species (BPP = 1.0, bootstrap = 66.8) *C. goytaca* (2n=54, FN=66) and *C. subflavus* (2n=54-56, FN=62-64) (Figure 7).

## Discussion

This is the most extensive study carried out in *Cerradomys* that integrates classical cytogenetics and chromosome painting within a phylogenetic framework.

Classic cytogenetic information obtained in this study agrees with previous chromosome data described for the eight *Cerradomys* species (Maia and Hulak, 1981; Almeida and Yonenaga-Yassuda, 1985; Langguth and Bonvicino, 2002; Tavares *et al.*, 2011; Bonvicino *et al.*, 2014), except for *C. akroai* in which a new fundamental number (FN=76) is described here, probably due to a pericentric inversion in one medium size acrocentric. In addition, pair 5 of *C. subflavus* was described as a homomorphic acrocentric (5a) or as a heteromorphic acrocentric/subtelocentric (5a5b) (Almeida and Yonenaga-Yassuda 1985), nevertheless the sample with 2n=56 herein reported showed pair 5 as a homomorphic subtelocentric (5b5b), a variation that has not been described previously.

It is worth mentioning that GTG-banding patterns are presented here for the first time in *C. marinhus*, *C. maracajuensis*, *C. akroai*, and *C. goytaca*. They were important as they confirmed accurately the location of the probes after chromosomal painting. Our results highlight that classical cytogenetic studies are still scarce in this group.

### Distribution of telomeric repeats

The patterns of distribution of telomeric repeats are presented here for the first time for *C. marinhus*, *C. maracajuensis*, *C. akroai* and *C. goytaca*.

The species that diverged early in the phylogeny (*C. marinhus* and *C. maracajuensis*)*,* together with *C. akroai* and *C. scotti*, presented telomeric signals restricted to the terminal regions of the chromosomes. Nevertheless, non-telomeric repeats (the so-called interstitial telomeric sites – ITS) were observed in *C. langguthi* and those species that belong to the most derived clade (*C. vivoi*, *C. goytaca*, and *C. subflavus*). Thus, the emergence of telomeric repeats occurred recently, since species that do not have ITS split from species that have ITS about 2.38 Mya (Tavares *et al.*, 2016).

Comparative analyses of the telomeric distribution and chromosome painting in *Cerradomys langguthi* revealed that from the five ITSs observed in pair 1, four coincide with sites of association between two OMO probes and one occurred in the middle of OMO 1. The pericentromeric ITS observed in the other pairs also occurred between two OMO probes. These results are in accordance with those reported by Nagamachi *et al.* (2013). ITS observed in *Cerradomys vivoi* corroborates the pattern described by Andrades-Miranda *et al.* (2002) and those observed in *Cerradomys goytaca* and *C. subflavus* occur at points of association between two OMO probes.

Our results, together with the molecular phylogeny and chromosome painting, suggests that the ITS observed in *Cerradomys* species are relicts of telomeres resulting from past fusions. In the case of CLA 1, multiple interstitial telomeric sequences resulted from *in tandem* fusions and in the case of CLA 3, CLA 4, CVI 1, CVI 4, CGO 1, CSU 1 and CSU 5/6, ITSs resulted from centric fusions.

Two different types of ITS have been described, according to their sequence organization and distribution: heterochromatic ITS (het-ITS) and short ITS (s-ITS) (Ruiz-Herrera *et al.*, 2008). Heterochromatic ITS are large stretches of telomeric sequences, localized mainly at pericentromeric regions and probably represent remnants of chromosomal rearrangements, while short ITS are few telomeric TTAGGG repeats localized at interstitial sites inserted during the repair of DNA double-strand breaks (Ruiz-Herrera *et al.*, 2008). The pericentromeric ITS observed in *Cerradomys* species, as well as the internal ITS associated between two OMO probes in *C. langguthi* probably belong to the het-ITS type while the ITS that co-localize to OMO 1 possibly belongs to s-ITS, showing that different mechanisms were responsible for the origin of TTAGGG repeats in this genus.

The non-telomeric sequences observed in the junction of pairs 1 and 3 of *C. langguthi* (CLA 2/7 and CLA 5/3, respectively) and pair 5/6 of *C. subflavus* are consistent with the hypothesis that het-ITS are unstable and prone to breakage since it is observed in nature samples with acrocentrics/subtelocentrics pairs CLA 2, CLA 7, CLA 3, CLA 5, CSU 5 and CSU 6 (Maia and Hulak, 1981; Almeida and Yonenaga-Yassuda, 1985; present study). In these cases, ITS can be acting as hotspots for chromosome rearrangements, conferring chromosomal plasticity to their holders (Ruiz-Herrera *et al.*, 2008; Bolzán, 2017).

Despite many vertebrate species showing ITS related to chromosome rearrangements (Meyne *et al.*, 1990; Lee *et al.*, 1993; Pellegrino *et al.*, 2004), non-telomeric repeat sequences have been observed also in species that present conserved karyotypes (Wiley *et al.*, 1992; Pagnozzi *et al.*, 2000; Metcalfe *et al.*, 2004). Alternative mechanisms by which non-telomeric repeats are generated include amplification of TTAGGG_n_ sequences, components of satellite-DNA, exchange, transposition or unequal sister chromatid exchanges introduced by telomerase or by transposons (Wiley *et al.*, 1992; Ruiz-Herrera *et al.*, 2008).

On the other hand, several species with highly rearranged karyotypes (detected by GTG-banding and chromosome painting), do not have ITS, suggesting that these sequences can also be lost by chromosome breakage (Silva and Yonenaga-Yassuda, 1997; Di-Nizo *et al.*, 2015). Although ITS were observed in *Cerradomys*, several rearrangements were detected without the presence of ITS (Figures 3 [Fig f4] [Fig f5]-6), showing that this genus underwent both retention and loss of ITS throughout its evolution, probably by chromosomal breakage, deletion or translocation of these sequences (Bolzán, 2017).

### Chromosome evolution within *Cerradomys* in the light of phylogenetic relationships

The cytogenetic results allied to the phylogeny provide a clear establishment of karyotype evolution in *Cerradomys*, showing that extensive chromosomal rearrangements are responsible for the karyotypic differentiation within the genus.

Only three out of the 20 OMO probes (OMO 25, 26 and 30) are conserved since they painted whole chromosomes in all *Cerradomys* species. The remaining probes show more than one signal in at least one species, revealing intense genome reshuffling in closely related species. Hypothetical rearrangements revealed by classical and molecular cytogenetics were plotted in the nodes of each clade and beside the lineages (Figure 7).

At least two fission events have occurred in the ancestor of the genus in addition to the association between probes OMO 4 and OMO 5 that can be considered as plesiomorphic, given that it was also observed in five *Oligoryzomys* species (Di-Nizo *et al.*, 2015).

Different rates of chromosomal changes were observed within *Cerradomys*. The clade composed of *C. marinhus* and *C. maracajuensis* is represented by more conservative karyotypes than its sister clade, in which extensive chromosome rearrangements are observed. Both species present the same diploid number (2n=56), but different fundamental numbers (FN=54 and FN=58, respectively). Comparative chromosome painting reveals similar hybridization patterns, corroborating the close relationship between them. In addition, the difference between the two fundamental numbers can be explained by pericentric inversions in two pairs: CMARI 1/ CMARA 1 and probably CMARI 25/ CMARA 2.

The remaining species (*C. scotti*, *C. akroai*, *C. langguthi*, *C. vivoi*, *C. subflavus* and *C. goytaca*) cluster in the sister clade and comparisons of chromosome painting and molecular phylogeny reveal that many rearrangements occurred during the evolution of these lineages.

Internal relationships show that *C. akroai* and *C. scotti* are closely related and that these species had experienced an increase in the diploid number, achieving the highest diploid numbers described for the genus. Although it was not possible to perform chromosome painting in *C. akroai* metaphases (2n=60, FN=76), comparative GTG-banding on the largest pairs suggest that the karyotype of *C. akroai* and *C. scotti* (2n=58, FN=72) differ by pericentric inversions in two medium pairs (CAK 1 / CSC 1 and CAK 2 / CSC 14) (not shown). In addition, a fusion/fission event plus at least two pericentric inversions or centromere repositioning, which could not be detected by GTG-banding comparison, are necessary to explain karyotypic differences between these species, showing that many chromosome changes occurred within this clade.

The next clade presents a decrease in diploid numbers, fission events as well as the presence of interstitial telomeric probes. Additionally, *C. langguthi* underwent one of the highest number of rearrangements leading to the lowest diploid number of the genus. Many rearrangements are also observed in *C. vivoi* (fissions, centric and *in tandem* fusions).

An increase in the diploid number is observed again in the next clade and the comparison between *C. goytaca* (2n=54) and *C. subflavus* (2n=56) shows that they are closely related, although complex rearrangements may be involved in their karyotype differentiation. It seems that a centric fission of pair CGO 3 or a centric fusion of pairs CSU 13 and CSU 6 (as described before, this pair is already involved in Robertsonian rearrangements within *C. subflavus*) leading to CGO 3, plus a paracentric inversion in one pair (probably CGO 14, CSU 12) and pericentric inversions in two small pairs (not detected by GTG-banding or chromosome painting) is required to differentiate both karyotypes. It is also worth mentioning that in *C. goytaca* and *C. subflavus* (2n=54), despite the same diploid number (2n=54), a much more complex scenario is required to explain the karyotypic differences between these two taxa.

Regarding OMO Xa, this probe paints the whole X in all species, as expected since this chromosome is highly conserved among placental mammals (Graves, 2006). The same probe also hybridizes to the euchromatic region of the Y that probably corresponds to the pseudoautosomal pairing region observed in other species of the tribe Oryzomyini (Moreira *et al.*, 2013; Di-Nizo *et al.*, 2015).

This work sheds light on the karyotype evolution of *Cerradomys*, and chromosome painting not only corroborates the GTG-banding pattern but also detects many more rearrangements. The rearrangements detected include *in tandem* and centric fusion, fission, centromere repositioning, and pericentric and paracentric inversions. Given the limitations of chromosome painting in detecting peri/paracentric inversions and reciprocal translocations and also that hybridizations were not possible with the entire chromosome set of *O. moojeni*, the chromosome evolution in *Cerradomys* is probably even more complex than observed.

According to Garagna *et al.* (2014), species with different chromosomal variants may be predisposed to form new species. This may be the case in *C. langguthi* and *C. subflavus*, since Robertsonian rearrangements and pericentric inversions were observed in both species (Maia and Hulak, 1981; Almeida and Yonenaga-Yassuda, 1985). Rieseberg (2001) proposed that this type of chromosomal rearrangement may not have a strong influence on fitness; instead, the suppression of recombination that leads to reduction in gene flow and to the accumulation of incompatibilities may fuel the process of speciation.

The occurrence and fixation of rearrangements can be surprisingly fast (Britton-Davidian *et al.*, 2000) and this would be the case in *Cerradomys* since very closely related species seem to have experienced huge and recent genomic reorganization, considering that the first speciation events in this genus was dated in the Pliocene and early Pleistocene (Tavares *et al.*, 2016). Thus, it is likely that the climatic oscillations of the Pleistocene played a role in the diversification of the genus, creating an ecological barrier to gene flow (Carnaval and Moritz, 2008; Tavares *et al.*, 2016).

### Comments on phylogenetic relationships and species status

As we obtained the phylogenetic reconstruction for *Cerradomys* in order to infer the trajectory of chromosome evolution, we can also observe that the topology obtained here corroborates almost all relationships among the species previously described in the literature (Weksler *et al.*, 2006; Percequillo *et al.*, 2008; Tavares *et al.*, 2011; Bonvicino *et al.*, 2014). Although some clades show relatively low supports, only *C. subflavus* has been recovered as paraphyletic in relation to *C. goytaca* so that they were considered as conspecifics by Bonvicino *et al.* (2014). However, more recently, morphological analyses show that they are distinct species (Tavares *et al.*, 2016). The cytogenetic data obtained in his work corroborate that *C. goytaca* is a valid species, since the complex chromosomal differences between this species and *C. subflavus* are compatible with reproductive isolation and hybrids may present meiotic problems due to mal-segregation and may not be viable. Furthermore, *Cerradomys goytaca* has a small effective population size and is geographically isolated from *C. subflavus*, occupying restricted areas of Restinga, a harsh and adverse environment (Tavares *et al.*, 2011, 2016). This may have facilitated the fixation of chromosome rearrangements.

## Supplementary material

The following online material is available for this article:

Table S1 - Specimens included in the molecular phylogeny with partial mitochondrial cytochrome *b* gene and sequences extracted from GenBank.

Figure S1 - Maximum likelihood (ML) phylogenetic relationships based on partial mitochondrial cytochrome *b* gene.
