# Supplementary material for: Extensive genomic reshuffling involved in the karyotype evolution of genus Cerradomys (Rodentia: Sigmodontinae: Oryzomyini)
Source: Genet Mol Biol. 2020 Nov 13;43(4):e20200149. doi: 10.1590/1678-4685-GMB-2020-0149 (PMC7783725; doi:10.1590/1678-4685-GMB-2020-0149)
Supplement: Supplementary file 1 [file 1415-4757-GMB-43-4-e20200149-s1.pdf]

Genetics and Molecular Biology
https://doi.org/10.1590/1678-4685-GMB-2020-0149

Supplementary Material to "Extensive genomic reshuffling involved in the karyotype evolution of genus *Cerradomys* (Rodentia: Sigmodontinae: Oryzomyini)"

Table S1 - Specimens included in the molecular phylogeny with partial mitochondrial cytocrome *b* gene and sequences extracted from GenBank. Taxa, GenBank Accession numbers, Field or Museum Number (when available) and localities in which samples were collected.

Taxon	GenBank #	Field or Museum #	Locality
C. marinhus	AF181279	MN63834*	Jaborandi, Bahia, Brazil
	KP122228	LBCE17294	Uberlândia, Minas Gerais, Brazil
	To be provided	**CRB1835**	**Cocos, Bahia, Brazil**
C. maracajuensis	AF181278	MN44178**	Maracaju, Mato Grosso do Sul, Brazil
	KP122224	CRB2790	São José do Xingu, Mato Grosso, Brazil
	To be provided	**MN71687**	**Parque Nacional Emas, Goiás, Brazil**
C. akroai	KP122214<br>KP122215<br>KP122219	MN80485<br>MN80486<br>MN80491**	Novo Jardim, Tocantins, Brazil
	To be provided	**MZUSP30347**	**Uruçuí-Una, Piaui, Brazil**
C. scotti	AF181277	MN50379	Cavalcante, Goiás, Brazil
	KP122232	LBCE10907	Aporé, Goiás, Brazil
	KP122234	LBCE17287	Campo Florido, Minas Gerais, Brazil
	To be provided	**MJJS189**	**Serra das Galés, Goiás, Brazil**
C. langguthi	AF181276	MN69789**	João Pessoa, Paraíba, Brazil
	KP122223	LBCE15905	Sousa, Paraíba, Brazil
	KR149696	PHA282	Paudalho, Pernambuco, Brazil
	To be provided	**JFV474**	**Piracuruca, Piaui, Brazil**
C. vivoi	KR149684	MN75905	Morro do Chapéu, Bahia, Brazil
	KP122249	MN61666	Juramento, Minas Gerais, Brazil
	AF181275	MN35898**	Itabuna, Bahia, Brazil
	KR149688	LG211	Itaetê, Bahia, Brazil
C. goytaca	KR149679	MN73261	São João da Barra, Rio de Janeiro, Brazil
	KP122221	LBCE-JCM10	Quissamã, Rio de Janeiro, Brazil
	To be provided	**NPM933**	**Restinga de Jurubatiba, Rio de Janeiro, Brazil**
C. subflavus	KP122247	LBCE17255	Uberlândia, Minas Gerais, Brazil
	To be provided	**CIT2053**	**Itirapina, São Paulo, Brazil**
	To be provided	**CIT1396**	**Rio Claro, São Paulo, Brazil**
Hylaeamys megacephalus (outgroup)	KP122250	LBCE18571	Not available
Neacomys amoenus (outgroup)	MG262329	CIT389	Aripuanã, Mato Grosso, Brazil

*Paratype. **Holotype. In bold, sequences obtained in this work. Collector and Lab or Museum acronyms: CIT (Banco de células do laboratório de Citogenética de Vertebrados, IB/USP), CRB (Cibele Rodrigues Bonvicino), JFV (Júlio Fernandes Vilela), MJJS (Maria José de J. Silva), PHA (Paulo Henrique Asfora), LBCE (Laboratório de Biologia e Parasitologia de Mamíferos Silvestres Reservatórios), MN (Museu Nacional, UFRJ, Brazil), LG (Lena Geise), MZUSP (Museu de Zoologia da Universidade de São Paulo) and NPM (Núcleo de Pesquisa em Ecologia e Desenvolvimento Sócio-Ambiental de Macaé).
