# Supplementary material for: Extensive genomic reshuffling involved in the karyotype evolution of genus Cerradomys (Rodentia: Sigmodontinae: Oryzomyini)
Source: Genet Mol Biol. 2020 Nov 13;43(4):e20200149. doi: 10.1590/1678-4685-GMB-2020-0149 (PMC7783725; doi:10.1590/1678-4685-GMB-2020-0149)
Supplement: Supplementary file 2 [file 1415-4757-GMB-43-4-e20200149-s2.pdf]

Genetics and Molecular Biology

https://doi.org/10.1590/1678-4685-GMB-2020-0149

Supplementary Material to "Extensive genomic reshuffling involved in the karyotype evolution of genus *Cerradomys* (Rodentia: Sigmodontinae: Oryzomyini)"

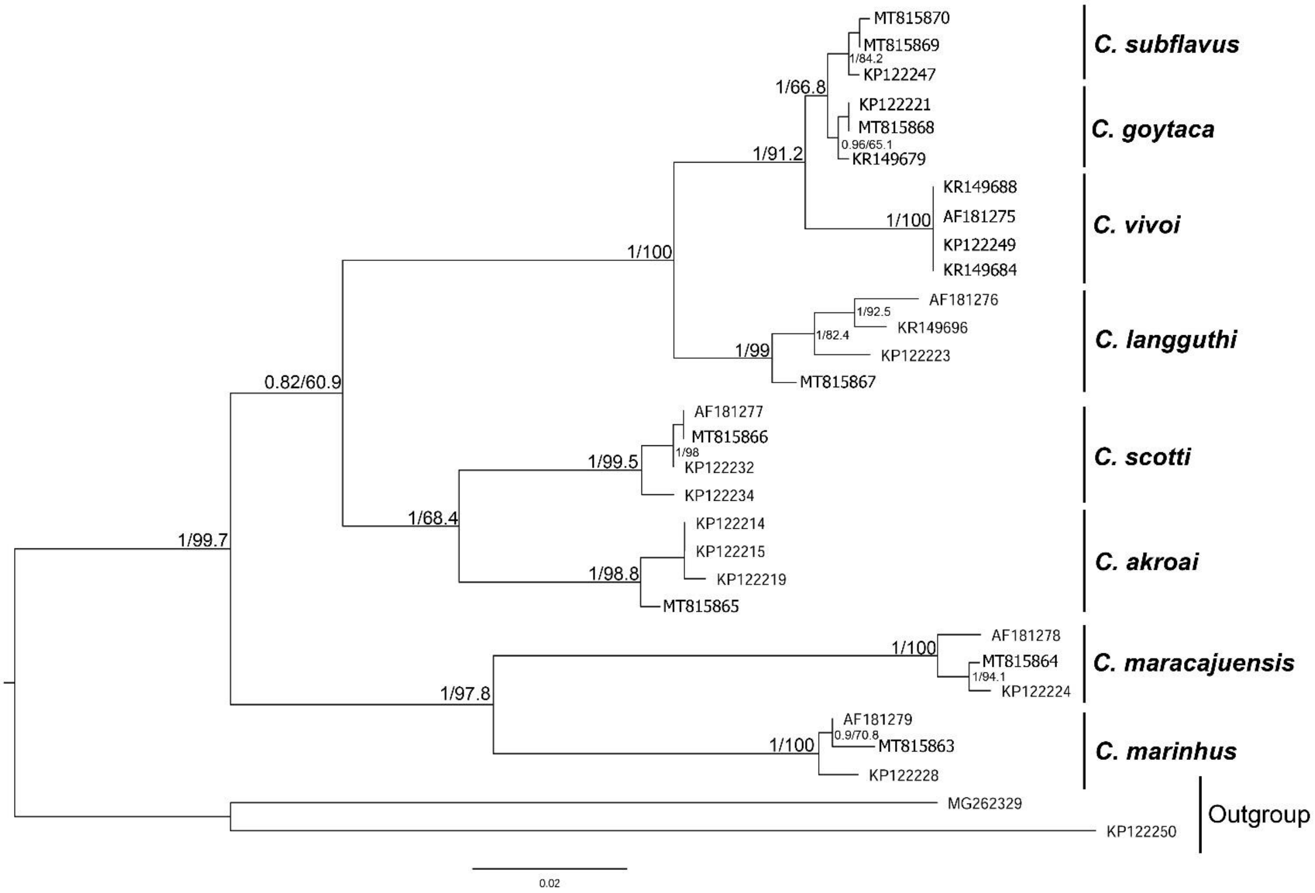

Figure S1 - Maximum likelihood (ML) phylogenetic relationships based on partial mitochondrial cytochrome *b* gene. Numbers above the nodes indicate Bayesian posterior probability and ML bootstrap, respectively.
